# Improving Breastfeeding by Empowering Mothers in Vietnam: A Randomised Controlled Trial of a Mobile App

**DOI:** 10.3390/ijerph17155552

**Published:** 2020-07-31

**Authors:** Thi Thuy Duong Doan, Colin Binns, Ngoc Minh Pham, Yun Zhao, Thi Phuong Hoa Dinh, Thi Thu Ha Bui, Trung Chuyen Tran, Xuan Hoai Nguyen, Roslyn Giglia, Fenglian Xu, Andy Lee

**Affiliations:** 1 Faculty of Social Sciences, Behavior and Health Education, Hanoi University of Public Health, 1A Duc Thang Street, Bac Tu Liem District, Hanoi 10000, Vietnam; dttd@huph.edu.vn (T.T.D.D.); phuonghoa55@yahoo.com (T.P.H.D.); bth@huph.edu.vn (T.T.H.B.); andy.lee@curtin.edu.au (A.L.); 2 School of Public Health, Curtin University, Bentley, WA 6102, Australia; minh.pn@tnu.edu.vn (N.M.P.); y.zhao@exchange.curtin.edu.au (Y.Z.); 3 Department of Epidemiology, Faculty of Public Health, Thai Nguyen University of Medicine and Pharmacy, Thai Nguyen 250000, Vietnam; 4 Faculty of Information Technology, Department of Hanoi University of Mining and Geology, 18 Vien Street-Bac Tu Liem District, Hanoi 10000, Vietnam; trantrungchuyen@humg.edu.vn; 5 Faculty of IT, Ho Chi Minh University of Technology (HUTECH), Ho Chi Minh 700000, Vietnam; nxhoai@hanu.edu.vn; 6 Foodbank Perth Airport, Perth, WA 6105, Australia; roslyn.giglia@foodbankwa.org.au; 7 Data Analysis & Surgical Outcomes Unit (DASO), Royal North Shore Hospital, St Leonards, NSW 2065, Australia; fenglian.xu@health.nsw.gov.au

**Keywords:** breastfeeding, maternal health, infant health, mobile application, Vietnam, CONSORT-SPI 2018

## Abstract

Breastfeeding provides benefits to the infant and mother; however, the rates of breastfeeding, particularly exclusive breastfeeding, remain below optimal levels in many Asian countries. The aim of this study is to review the benefits of breastfeeding to mothers and infants and current rates of breastfeeding in Vietnam, and to evaluate the effectiveness of a mobile application on exclusive breastfeeding among mothers in Vietnam. A two-arm, parallel triple-blinded randomised controlled trial will be conducted among 1000 mothers in Hanoi City, Vietnam, during 2020–2021. Eligible participants are pregnant women who will seek antenatal care from health facilities at 24–36 weeks of gestation and plan to deliver at two participating hospitals, own a smartphone, and carry a singleton foetus. Permuted-block randomisation method stratified by maternal age, education and parity will be used to ensure an equal number of participants in each group. A smartphone app will be developed to deliver breastfeeding and non-breastfeeding information to the intervention and control group, respectively. Data will be collected at baseline, before hospital discharge, and at 1, 4, and 6 months postpartum. This study envisages demonstrating whether a smartphone-based intervention can be effective at improving breastfeeding in Vietnam. Trials registration: ACTRN12619000531112.

## 1. Introduction

The benefits of breastfeeding for infants, mothers, global health, and the environment are substantial, and are well-documented [1,2,3]. Increasing breastfeeding rates will be an important factor in achieving the sustainable development goals [4].

The World Health Organization (WHO) recommends breastfeeding a newborn within one hour after birth (early initiation of breastfeeding) and, exclusively breastfeeding the infant for the first six months. Exclusive breastfeeding is defined as “giving only breastfed milk, without any other food or liquid, even water” [5]. According to The United Nations Children’s Fund (UNICEF), the worldwide rate of early initiation of breastfeeding is estimated at 44% and exclusive breastfeeding for the first six months was reported as 40% [6,7]. The World Health Organization (WHO)/UNICEF define exclusive breastfeeding as breastfeeding, without any additional food or fluids, not even water, for the first six months, but in practice it is measured as prevalence in the previous 24 h in a cross sectional survey [8]. This means that actual exclusive breastfeeding rates (EBF) are lower than the figures obtained from cross-sectional data [9]. 

### 1.1. The Benefits of Breastfeeding and the Current Rates of Breastfeeding in Vietnam

There are numerous benefits to infants who are breastfed in the short and longer term. The WHO Collaborative Group found a substantial increase in infant mortality in the first few years of life in infants who were not breastfed, from infection and all ‘causes’ [10]. There are around 5 million deaths annually of children under 5 years of age and it is estimated that increasing breastfeeding rates to recommended levels could save 823,000 children’s lives annually [1]. A recent review confirmed the importance of breastfeeding in protecting against many infections; compared to infants given formula, there are significantly lower rates of diarrhoeal disease and lower respiratory tract infection, with a reduction of 50% or more to be expected, especially in infants under six months of age [11,12]. Exclusive breastfeeding is important for establishing a healthy microbiome, which has an important protective effect against chronic disease later in life, including obesity [13,14,15]. Children who had been breastfed for at least six months were less likely to be overweight or obese later [16]. Breastfeeding is associated with lower rates of chronic illness, including obesity, diabetes, hypertension and obesity [1,17,18,19]. Moreover, breastfeeding is associated with improved cognitive outcomes for infants and can prevent them from adverse health effects of air pollution [20,21]. 

Mothers also benefit substantially from breastfeeding. Short-term benefits to mothers include reduced post-partum haemorrhage and infection, lactational amenorrhea, increased rate of weight loss, reduced adiposity, lower rates of post-partum depression, and reduced stress and anxiety [22,23,24]. The long-term benefits of breastfeeding to mothers have been summarised in several reviews [1,17,25]. The benefits of breastfeeding include reducing the rates of breast and ovarian cancer, hypertension, diabetes and post-partum depression and there will be less retention of weight post-delivery, resulting in the reduction of preventable disability-adjusted life years (DALYs). It is estimated that increasing rates of breastfeeding would prevent 20,000 breast cancer deaths annually. Reviews of the impacts of insufficient breastfeeding in mothers from Mexico and Brazil have shown cost-savings that run to billions of dollars [24,26]. 

Mothers who breastfeed have reduced rates of ovarian cancer proportional to the total length of breastfeeding [27]. There are reductions in the rates of breast cancer [28,29,30]. Obesity is also less in breastfeeding mothers; on average, obese mothers who breastfed are 8 kg lighter six years after delivery compared to mothers who had not breastfed their infants [31]. Type 2 diabetes, hypertension, and hyperlipidaemia are also less, although these effects could, at least in part, result from reductions in obesity rates [32,33]. A large meta-analysis of cohort studies reported an approximately 30% significantly lower risk of maternal type 2 diabetes comparing mothers with the longest duration of breastfeeding versus those the shortest duration [34]. Women with gestational diabetes mellitus (GDM) and breastfed for longer than 6 months had the lowest risk of continuing type 2 diabetes, compared to those did not breastfeed [35]. Longer duration of lactation is also associated with a lower risk of type 2 diabetes among women with a history of GDM [36]. This is important for Vietnam which like many low and middle-income countries (LMICs) has increasing rates of GDM and postnatal type 2 diabetes [37]. There may be an overall reduction in risk for cardiovascular disease proportional to breastfeeding duration [38]. The Women’s Health Initiative Study (*n* = 139,681) reported that a lifetime history of more than 12 months lactation was associated with lower risks of cardiovascular disease, diabetes, hypertension, hyperlipidaemia, and hypertension as compared to women who never breastfed [39,40]. The EPIC cohort study (*n* = 322,972) found that mothers who had breastfed an infant had around 20% significantly reduced risk of dying during the following decade [41].

UNICEF considered the potential impacts of breastfeeding and summarised in this way “In short, breastfeeding is among the most effective ways to protect maternal and child health and promote healthy growth and optimal development in early childhood. Empowering and enabling women to breastfeed should be at the heart of countries’ efforts to keep every child alive and to build healthy, smart and productive societies” [8].

The East Asia and the Pacific Regions have the lowest rates of early initiation of breastfeeding (36%) and reported period prevalence (0–6 months) of exclusive breastfeeding (27%) [42]. In 2019 UNICEF reported exclusive breastfeeding rates (<6 months) of 24% in Vietnam, early initiation of breastfeeding 26%, continued breastfeeding (12–23 months) 43%, including 55% in the poorest quintile and 29% in the richest quintile [43]. The early initiation rate has declined from 74% in a 2004 study to the present low level of 26% [44]. In this study, mothers who delivered at home had an early initiation rate of only 24%. By week 16 the exclusive breast feeding had dropped to 44% and no-one was exclusively breastfeeding at 6 months [45]. In 2016, only 40% of infants had “early initiation” of breastfeeding, while 17% of infants aged 0–5 months received only breast milk during the previous day (i.e., period prevalence 0–6 months) [46]. The exclusive breastfeeding rate at 6 months was less than 1% [47]. In a large cohort study (*n* = 1709), the prevalence of prelacteal feeding was high (56.5%) and formula feeding was common (79.5%) before hospital discharge. This increased the risks of hospitalisation by 2 months for prelacteal feeds and infant formula use [48]. 

In Vietnam, it has been projected that improved breastfeeding rates would prevent between 2000 and 8000 infant deaths annually and around 500 mothers dying from breast cancer [1,49]. Although increases in breastfeeding rates have been observed in some developing countries [50], the breastfeeding prevalence continues to decline in Vietnam, particularly in urban areas [46,51] and following caesarean delivery [46,47]. However, the surveys reported used different methodologies and the review by Cai used 24 h recent feeding in a cross-sectional survey and the results completely depend on the age of the infants being surveyed [50].

### 1.2. Barriers to Breastfeeding Practices in Vietnam

A conceptual framework, to explain barriers of early breastfeeding initiation, duration, and exclusive breastfeeding proposed three levels of social influence: Social-political, group, and individual [52]. These three levels are evident in breastfeeding policy in Vietnam and while the government has endorsed policies to support breastfeeding, commitment and enforcement to these policies is lacking [53,54]. Breastfeeding is part of the national strategy on nutrition, but resources assigned for breastfeeding promotion activities remain limited [55]. Fewer than 9% of hospitals met the standards of the Baby-Friendly Hospital Initiative [49]. The advertisement and promotion of breastmilk substitutes has been banned since “Decree 21” in 2006, and “Decree 100” in 2014, but advertisements for formula milk are still appearing in the media [56,57]. Despite the extension of maternity leave to 6 months, less than 36% of mothers received maternity leave paid by social insurance in 2017 [58].

Group-level factors in the environment include health facilities, households, workplaces, and communities [52]. In health facilities, caesarean section usually results in prelacteal feeding [59]. Like most LMICs, the rate of caesarean sections in Vietnam is increasing and is reported to be 40–60% in public in 70% in private hospitals [60,61]. Knowledge, attitudes, and skills of health professionals about breastfeeding remain limited [55]. Only half of the pregnant women received breastfeeding advice during pregnancy and one-third during their hospital stay [57].

Social support for exclusive breastfeeding from community/family members was also low [57,59,62,63]. Grandmothers generally support ‘any breastfeeding’ but not ‘exclusive breastfeeding’ [64,65]. Meanwhile, Vietnamese mothers often consider yellowish colostrum as “false” milk and perceive formula milk to be more ‘pure’ than human milk [66]. Feeding a newborn with formula milk and water is practised by family members to assist mothers in recovering after giving birth [57]. In a review of complementary food introduction in Asia, Vietnam was in the lowest group of countries with a median age of 4 months [67]. Mothers often introduce complementary foods early because of their perception that breastmilk alone cannot meet the needs of their children [68].

Individual-level factors refer to characteristics of mothers, infants, and the ‘mother-infant dyad’ [52]. Vietnamese women believe that breastmilk has enough nutrition for their babies and had the confidence to breastfeed [69,70]. Indeed, over 70–86% of mothers intended to practise exclusive breastfeeding [71,72]. Despite good intentions, discarding colostrum and feeding a newborn with water and honey are not uncommon [59,62,66,69]. Formula milk is introduced whenever the mother’s milk supply is believed to be insufficient, especially in the presence of crying [71,73]. Mothers would undertake exclusive breastfeeding if they have better knowledge about its benefits, have beliefs and confidence in the ability of breastfeeding, receive advice from “public information”, gain support from health professionals and family members on exclusive breastfeeding [59,63]. Several breastfeeding problems experienced by mothers have been reported in Vietnam, including breast problems (e.g., cracked nipples, breast engorgement, mastitis), insufficient child suckling or attachment problems, insufficient lactation and postpartum fatigue [57,64,71].

### 1.3. Community-Based Interventions to Improved Breastfeeding Outcomes

To overcome these barriers, several interventions for improving exclusive breastfeeding have been implemented in Vietnam. The Alive & Thrive program was implemented in Vietnam between 2009 and 2014 to improve infant feeding practices with an intervention package that included counselling, mass media, and community mobilisation [74,75]. A cluster-randomised, non-blinded study involving 2000 participants was designed to evaluate its effectiveness. After the intervention, the period prevalence of exclusive breastfeeding until 6 months increased from 18.9% to 57.8% in the intervention group [74]. Early initiation of breastfeeding, however, declined from 60% to 53%. This decline was less than that of the control group [74]. 

In another intervention, breastfeeding information was provided to the husband during pregnancy, delivery, and postpartum either at a health facility or home, who also participated in fathers’ clubs to discuss breastfeeding with their peers. A quasi-experimental study was designed to evaluate the main outcomes of 802 couples in 2013 [76]. After the intervention, the early breastfeeding rate in the intervention area (81.2%) was double that of the control area (39.6%) [77]; while exclusive breastfeeding (16.0%) was also significantly higher in the intervention group when compared to the control group (3.9%) [78]. 

In a pilot study, the Ministry of Health provided the “Maternal and Child Health Handbook” to 810 pregnant women, followed by face-to-face verbal guidance in four provinces. The intervention, despite without a control group, resulted in an increase in exclusive breastfeeding rate from 18.3% to 74.9% [79]. However, only 68.1% of the recorded information related to exclusive breastfeeding [79]. 

None of these studies used a mobile Health (mHealth) based intervention or targeted women living in an urban area. Mobile Health plays a role in breastfeeding promotion. The World Health Organization defines mHealth as “the use of mobile wireless technologies for public health” [80]. For breastfeeding education, although counselling is effective [81], it is time consuming, costly, and difficult to implement. Recent systematic reviews and meta-analyses found that mHealth interventions improved exclusive breastfeeding in high-income and LMIC countries [82,83,84,85]. Recent effective mHealth interventions on exclusive breastfeeding in the United States [86], Australia, China [87,88,89], and Nigeria [90] used the website, e-mail, online chat, webcam, text messages, and/ or phone call in conjunction with long postnatal support [82]. For example, a recent study showed that notifications were the most effective means of motivation for the Milkman app [91]. Sending one weekly text message significantly improved exclusive breastfeeding rate at six months, from 6.3% to 15.1% in Shanghai, China [88]. However, three of the five randomised controlled trials mentioned above intervened using mHealth only [86,88,89], while two others integrated mHealth as one component of the intervention strategies [87,90]. 

Mobile-based solutions include voice, text messages, and/or websites. Recent solutions have shifted to more sophisticated mobile applications [92]. A mobile application can provide text messages in the form of notifications, information on the most appropriate infant feeding, and encouragement for health behaviour changes. Other interactive features include “finding a suitable public place for breastfeeding” and tracking the time and duration of breastfeeding [92]. However, most applications on infant feeding have been rated as poor quality [93], lacking evidence-based recommendations, and without a rigorous evaluation of its effectiveness [94]. In Australia, several mobile applications to support breastfeeding for women have been well accepted in rural areas and have been resulted in increasing exclusive breastfeeding rates [89,95]. A quasi-experimental study on the “Growing Healthy mHealth Program” found no differences in exclusive breastfeeding rates between the intervention and control groups [96]. Other studies have focused on promoting exclusive breastfeeding through fathers [97,98,99]. Mobile applications have also been developed in other countries such as Thailand [92] and the United States [100,101,102], which have focused on the feasibility and acceptability of the mobile application, though more studies on their effectiveness are needed [103,104]. 

In Vietnam, mass media is the main source of information on breastfeeding, particularly among mothers living in big cities. Reliable resources on breastfeeding are lacking. Breastfeeding information can be found on the official websites of the Ministry of Health, the National Institute of Nutrition (NIN), and several large obstetric hospitals. However, except for the NIN website, these websites have recorded only a limited number of visits [105]. 

Unofficial sources of information, e.g., Facebook, are active in providing and sharing information on infant feeding. A study in 2015 suggested that young Vietnamese people in urban areas spent an average of 3 h per day on Facebook. About 72.9% of them were interested in health information shared on Facebook, and more than 50% thought Facebook-shared information was reliable [106]. A breastmilk group on Facebook has recruited 247,723 members since 2013 and has both online and offline activities to support and advocate for breastfeeding, but as there is no quality control it can also include false and controversial information.

Similar to other countries with limited resources, exclusive breastfeeding is important for public health in Vietnam, so that the use of mHealth may offer a practical solution to maintaining an effective and sustainable intervention program. A randomised controlled trial of a mobile application to support exclusive breastfeeding, including mothers who deliver by caesarean section, will be undertaken in Vietnam. 

The objective of this study is to empower mothers by providing them with information and motivation to exclusively breastfeed their infants, especially for those who have a caesarean section. This protocol describes a randomised controlled trial to evaluate the effectiveness of the breastfeeding mobile application for improving the exclusive breastfeeding rates in the trial group when compared to the control group, at 1, 4, and 6 months postpartum.

## 2. Methods 

### 2.1. Trial Design

**Figure 1:**
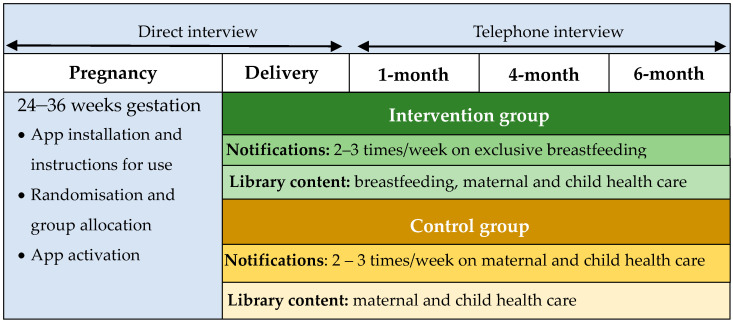
Study design and interventions.

This is a two-arm, parallel triple-blinded randomised controlled trial designed to compare exclusive breastfeeding rates and duration between the intervention and control group. Notifications and information will be provided to both groups through a smartphone mobile application. The intervention group will receive notifications mainly related to breastfeeding, whereas the control group will receive notifications on maternal and infant care only. The pregnant women recruited will be randomly assigned to either the intervention or control group on a 1:1 ratio (see below for details). The trial was developed according to the guidelines for Standard Protocol Items: Recommendations for Interventional Trials (SPIRIT) statement. The research protocol is reported following the requirements of the “Guidance for Reporting Social and Psychological Intervention Trials CONSORT-SPI 2018”(Consolidated Standards of Reporting Trials for Social and Psychological Intervention) [107]. The study design is illustrated in a Figure 1.

### 2.2. Participants

The study is conducted in Hanoi City, Vietnam. Hanoi is the capital of Vietnam and the second-largest city by population (7.4 million inhabitants in 2017) [108]. Breastfeeding rate within one hour of birth was low at 45.2%, while exclusive breastfeeding rate within the three first days was only 25.8%. The rate of predominant breastfeeding until the first 6 months was 12.6%, lower than the national level [109]. In Hanoi, the internet was the second most popular source of information mothers looked for and it had increased exponentially from 21.8% to 41.8% within the period 2012–2014 [109]. This presents an opportunity to implement an intervention using a mobile application, especially because of the high smartphone usage in the major cities of Vietnam (84%), including Hanoi [110].

We will recruit mothers who intend to deliver in either at Dong Anh general district hospital or Hanoi Gynaecology and Obstetrics hospital. The Dong Anh general district hospital is a typical hospital in a suburban district of Hanoi. The hospital had 6000 deliveries per year, 41% of which were caesarean sections. The Hanoi Obstetrics and Gynaecology hospital serves its nearby residents in the city centre, and assists an average of approximately 44,000 deliveries per year, with around 53% undergoing caesarean sections.

Mothers can be included if meeting the following criteria:

- attending antenatal clinics at 24–36 weeks of gestation;
- own a smartphone with at least:
  - iOS version 11 (announced from 2017, support for iPhones from 2015)
  - Android 5 (released from 2014);
- carrying a singleton foetus.

Mothers will be excluded if they meet any of the following criteria:

- being referred from other hospitals for high-risk pregnancy treatments;
- receiving advice from doctors against breastfeeding because of her health condition (e.g., positive T-cell lymphotropic virus, untreated brucellosis, varicella, H1N1 influenza) [111];
- where an infant is born with a birth weight of less than 2500, or is admitted to neonatal intensive care unit, the mother will continue receiving information from the app, but will be excluded from the analysis;
- if the infant has any of the infections or metabolic conditions as advised by the WHO or the Ministry of Health, where breastfeeding is contraindicated [11,112].

### 2.3. Intervention

Messages and library resources for the intervention and control groups will be developed separately and delivered to participants through a smartphone app. The design, development and evaluation of the app will be adapted from previous studies on mHealth and infant feeding [91,96,99,113,114,115,116,117]. 

Design. The mobile app will be designed for both iOS and Android platforms. Health education materials will be distributed to mothers by notifications and via access to an information library. Age-relevant notifications will be released two or three times per week to participants. The notifications will convey the key messages for improving breastfeeding, maternal, and child health care. Further information can be found by browsing records of the information library. The library records will list frequently asked questions together with answers. Sources of information and external links for further reading will be included in each record. When a participant touches the notification on her mobile screen, the app will automatically open and link to the relevant record in the library. Participants can retrieve notifications and search information in the app at any time (both online and offline modes). 

Content. The breastfeeding messages will be developed following the COM-B system (capability, opportunity, motivation, and behaviour) and Social Cognitive Theory as the underlying theoretical frameworks [118]. The mother’s capability refers to knowledge about the benefits of breastfeeding, and skills in breastfeeding. Opportunity includes support provided by health professionals or family members on breastfeeding. Motivation refers to a desire to perform the behaviour, forming plans and goals of early initiation of breastfeeding and exclusive breastfeeding [96]. Because of the importance of social norms, notifications will be targeted based on caregivers’ attitudes toward breastfeeding [119,120]. Notifications and relevant records on breastfeeding will be released to the intervention group to empower mothers through information and motivation:

- establish beliefs on the benefits of exclusive breastfeeding until 6 months;
- enhance the perception of sufficient breastmilk, for both vaginal delivery and caesarean section mothers;
- plan/commit to breastfeeding exclusively from birth;
- benefits and feasibility of early initiation of breastfeeding;
- overcome difficulties of breastfeeding; able to breastfeed without pain/problems;
- encourage mothers to seek advice and maintain communication with health professionals;
- involve husbands and grandmothers in preparation for exclusive breastfeeding;
- ask friends and relatives not to bring gifts of infant formula to the hospital or to the home.

Breastfeeding messages will not be released to the control group. The control group will receive notifications and library information on maternal and child health care only (see Figure 1). Relevant health education materials and guidelines from the Vietnam Ministry of Health, the National Institute of Nutrition, World Health Organization, infant feeding guidelines for the Asia Pacific Region, and the Australian government on infant feeding will be adapted to develop the library contents [25,121].

### 2.4. Outcomes

To evaluate the effectiveness of the mobile app, data will be collected five times from all participants: At enrolment, before discharge from hospital, 1 month, 4 months, and 6 months postpartum. The first two data collections will be conducted by face–to–face interviews and the three follow up surveys will be performed through telephone conversations. Definitions of breastfeeding from the World Health Organization (2008) will be adopted for the evaluation of the outcomes [5], based on both recall of the previous day and recall since birth methods [5,122,123]:

- early initiation of breastfeeding: Proportion of infants at the first month of age who were put to the breast within one hour of birth;
- exclusive breastfeeding: Proportion of infants at 1, 4, 6 months of age who were fed by breast milk only during the previous day, and from birth;
- predominant breastfeeding: Proportion of infants at 1, 4, 6 months of age who received breast milk as the predominant source of nourishment during the previous day, and from birth;
- any breastfeeding: Proportion of infants at 1, 4, 6 months of age who were ever breastfed;
- duration of exclusive breastfeeding: The age in weeks when infants received only breast milk from birth;
- introduction of complementary and solid foods: Proportion of infants at 1, 4, 6 months of age who received solid, semi-solid, or soft foods.

Confounding variables will be identified from an extensive literature review, including: (1) Socio-demographic characteristics such as age, marital status, education level, occupation, household income, parity, smartphone usage experience; (2) obstetric and birth outcomes: Complications during and after pregnancy, mode of delivery, length of hospital stay, infant gender, birth weight and length; (3) breastfeeding attitude and intention on breastfeeding. Such information will be collected prospectively from the baseline and follow up surveys.

### 2.5. Sample Size

The Clinical Calculators ClinCalc program is used for sample size estimation [124] following the formula for statistical superiority design with a dichotomous variable [125]. Among the above outcomes, the period prevalence of exclusive breastfeeding at 6 months is selected for sample size calculation: 12.6% in Hanoi in 2014 [109]. Assuming the prevalence to increase to 20% in the intervention group, the required sample size is 780 (390 per group) with 80% power at 5% level of significance. 

After accounting for a potential 15% loss of follow up from pregnancy to delivery and further 1–2% at each time point of the follow-up period, a final sample size of 1000 women will be recruited at baseline. We anticipate a high loss of follow up the rate at the initial stage due to mothers changing their minds and delivery at a different hospital and the possibility of incurring medical conditions or pregnancy complications that prevent breastfeeding. This sample size of 1000 allows about 20% loss of follow up for evaluation at 6 months [126]. Participants will be consecutively recruited from the two hospitals until reaching the desired sampling size.

## 3. Randomisation and Masking

### 3.1. Randomisation Procedure

Permuted-block randomisation method with block sizes of 2, 4, and 6 will be used with stratification based on age (<30 vs. ≥30 years), education level (<tertiary education vs. ≥tertiary education) and parity (<2 vs. ≥2 children) to achieve the balance of the number of participants allocated to each treatment group [127,128]. These three factors were selected because they are important prognostic factors of breastfeeding outcomes [129,130].

### 3.2. Sequence Generation

The randomisation list will be generated by an independent statistician using the Stata “ralloc” procedure with the randomisation-stratified scheme described above (StataCorp, College Station, TX, USA). The randomisation list with a 1:1 allocation ratio will be configured into the Research Electronic Data Capture (REDCap) software hosted at Curtin University in Perth, Australia. REDCap is a secure, web-based software platform designed to support data capture for research studies, providing (1) an intuitive interface for validated data capture; (2) audit trails for tracking data manipulation and export procedures; (3) automated export procedures for seamless data downloads to common statistical packages; and (4) procedures for data integration and interoperability with external sources [131,132].

### 3.3. Allocation Concealment Mechanism

A statistician of the research team will undertake randomisation using REDCap following the completion of baseline interview for each participant, and group allocation will be conducted via a website (https://vbf.geomatics.vn/) to activate the participant’s mobile app based on a unique identifier. Participants, research assistants and data analysts will be kept blinded to group assignments.

### 3.4. Implementation

Research assistants (data enumerators) from the Hanoi University of Public Health will recruit and enrol pregnant women who attend antenatal care clinics. They will invite eligible women to participate in the study, collect consent forms, help with downloading, installation, and provide guidance on using the mobile app. They will also interview consenting participants at the baseline survey. After the mobile app is activated, notifications and library information will be automatically sent to participants according to their assigned group (intervention or control). After delivery, the app will be updated to provide appropriate messages. Research assistants will follow up mothers by telephone interview at 1, 4, and 6 months postpartum.

### 3.5. Analytical Methods 

Data will be collected and managed using REDCap [131] and exported to Statistical Package for the Social Sciences, SPSS. Survival analysis will be used to compare the exclusive breastfeeding duration between the intervention and control groups. The Kaplan–Meier curves will be used for estimating the median exclusive breastfeeding times, and the log-rank test will be used to assess the differences between groups. Multivariable logistic regression and repeated measures mixed modelling will be applied to determine the effect of the intervention on exclusive breastfeeding prevalence at different time-points, accounting for delivery method, and other confounding factors. Intention-to-treat analysis will be performed in the presence of missing data to accommodate the expected attrition and withdrawal of participants from the study. All statistical analyses will be performed in SPSS (IBM Corp. Released 2017. IBM SPSS Statistics for Windows, Version 25.0. Armonk, NY, USA: IBM Corp).

### 3.6. Ethical Issues

The study protocol has been approved by the Curtin Human Research Ethics Committee (Ref: HRE2019-0143) and the Ethical Review Board for Biomedical Research, Hanoi University of Public Health (Ref: 28/2019/YTCC-HD3). 

Potential participants will be invited to take part in this ‘maternal and child health’ study. After briefing them about the purpose and research procedure, they will be asked to sign the written consent form in Vietnamese. Participants are free to withdraw at any time or may decline to answer any question at the baseline and follow up surveys without prejudice. Any problem or difficulties will be referred to health professionals. All participants and their infants, irrespective of their group allocation, will receive standard health services and hospital care. There are no potential risks for the participants involved in the study. All participants will be de-identified before data collection. Survey data captured through REDCap will be password protected and stored at the Curtin secure server, and accessible by the Curtin project manager and Chief Investigators only. Findings will be presented in an aggregate format in subsequent publications.

## 4. Discussion

Breastfeeding rates, particularly exclusive breastfeeding, are declining in Vietnam and almost all the rapidly developing countries in the Asia Pacific region. If breastfeeding rates can be improved there will be many benefits to short and long term health outcomes [133]. It is important to reverse this trend and increase the rates in order to improve the health of the population, and to assist in achieving the United Nations (UN) Sustainable Development Goals and improving the environment [134]. The costs of not breastfeeding are a significant drain on health services and the national economy [135,136].

The proportion of mothers in Vietnam who have access to a smartphone has now reached above 90%. There are 145 million active mobile phone connections for a population of 97 million [137]. We hope to achieve our outcomes given mobile apps increasingly being applied to improve maternal and infant health [138]. We anticipate that the widespread use of smartphone will offer great potential for implementing interventions to promote exclusive breastfeeding in Vietnam.

### Strengths and Limitations

This trial hypothesizes that there will be higher rates and longer duration of exclusive breastfeeding for up to 6 months after childbirth in the intervention group compared with its control counterpart. The study, to our knowledge, will be the first mobile app-based intervention to support breastfeeding in Vietnam. A blinded randomised control trial that can provide the most rigorous evidence of intervention effectiveness. The intervention content will be developed by experts in the field, and an app will be designed for two common mobile operating systems (iOS and Android). Data collection and management will be undertaken in a secured environment through REDcap so that data quality and protection can be ensured. The study will fill an important gap by developing a novel mHealth intervention addressing suboptimal breastfeeding in LMICs.

However, the study has some limitations. The intervention and control group may differ in terms of socio-demographic characteristics, potentially limiting comparison between the two groups on breastfeeding outcomes. Nonetheless, a statistical analysis plan will be developed, including adjustment for baseline covariates [139,140,141]. In fact, the CONSORT statement recommended against performing significance testing of baseline differences in randomised controlled trials [142]. Moreover, a stratified permuted-block randomisation will be undertaken with consideration of three key factors (i.e., maternal age, education and parity) associated with breastfeeding behaviours, perception and practices. Loss to follow-up is also a matter of concern because it may bias the results [143], particularly if missing data is associated with breastfeeding. To cope with this potential problem, we will adopt different strategies to maintain a high level of participant retention such as building positive rapport between participants and the study team, sending reminders, providing incentives, etc. [144,145,146]. In addition, appropriate methods will be applied to handle missing data, including inverse probability weighting, likelihood-based methods, and multiple imputation [147,148]. Another possibility is no or low usage of the mobile app under the study, but we will send reminders to encourage participants and conduct intention-to-treat analysis accordingly.

## 5. Conclusions

Breastfeeding has many advantages for mothers and infants in the short term and for lifetime health. The coverage in Vietnam of mobile phones is now above 90% making it eminently feasible to use smart phones for promoting breastfeeding. The app will be developed to promote early initiation of breastfeeding and exclusive breastfeeding for the first six months of life. In particular, the app will seek to overcome the low rate of breastfeeding following caesarean section.
